# Bibliometrics of orthopaedic articles published by authors of Germanophone countries

**DOI:** 10.1007/s00264-021-05052-y

**Published:** 2021-05-03

**Authors:** Ioannis Stratos, Marius M. Scarlat, Maximilian Rudert

**Affiliations:** 1 Department of Orthopaedic Surgery “Koenig-Ludwig-Haus”, Julius-Maximilians University Wuerzburg, Brettreichstrasse 11, 97074 Wuerzburg, Germany; 2 Clinique Chirurgicale St Michel, Groupe ELSAN, Toulon, France

Scientific publications are an important part of our professional environment, used as a measurement for academic productivity. They highlight success in medical research and also in other major fields of activity [1, 2]. In regard to the number of orthopaedic publications, some countries can be identified as major contributors. The United States are generating the highest volume of orthopaedic scientific articles per year worldwide, and the European countries contribute by the second largest number of articles [3]. Germanophone countries (Germany, Austria, and Switzerland) account for the highest quantity of Orthopaedic research articles within Europe [3]. The volume of surgeons and need for communication within the national society generated one of the most prominent non-English specialty Journal named “Der Unfallchirurg”, from 1894-1986 “Monatsschrift für Unfallheilkunde”,  and published by Springer with a tradition of 124 years and an impressive list of authors, correspondents, and referents. Other non-English specialty Journals, such as the French “Revue de Chirurgie Orthopédique” or the Italian Journal “Minerva Ortopedica e Traumatologica” switched to English for communicating research through a more visible platform. However, German-speaking scientists communicate very well in German as well as in English expression Journals. Therefore, the papers published in “International Orthopaedics” represent a small part of the volume of research published by German-speaking colleagues.

Despite the open accessibility of bibliographic data, little is known about the bibliometric characteristics of orthopaedic articles originating from Germanophone countries. Therefore, we conducted a bibliographic analysis using the database “Web of Science Core Collection” for International Orthopaedics (IO) on November 24, 2020. All published articles in the IO were identified for the period 2017 and 2018 (n = 766), grouped, and statistically analyzed (Table 1). Comparisons were performed between Germanophone countries (Germany, Austria, and Switzerland; GER; n = 94) vs. all other countries (non-GER; n = 672). By analyzing the country of origin for GER publications, we could identify 62 publications from Germany, 16 from Austria and 21 from Switzerland. Subgroup analysis for the “document type” did not show any difference between GER and non-GER publications (original articles vs. reviews vs. other articles; chi-square; p = 0.098 for at least one author from GER and p = 0.078 for corresponding author from GER). Further subgroup analysis classified 59 GER publications that were directly or indirectly associated with patient outcomes (clinical studies).

**Table 1:** Summary of the major categories and subcategories. Data are given in percent of total (%) and count (in parenthesis)

document type		
Article: 78% (594)	Review: 13% (98)	Other: 10% (74)
country of any author		
GER: 12% (94)	non-GER: 88% (672)	
country of the corresponding author		
cor-GER: 11% (85)	cor-non-GER: 89% (681)	
number of citations in all databases per year		
2.3±0.1		
anatomical interest of the article		
Spine: 10% (75)	Pelvis: 2% (19)	Foot & Ankle: 6% (44)
Shoulder & Elbow: 13% (101)	Hand & Wrist: 2% (14)	Long Bones: 3% (23)
Hip: 32% (244)	Knee: 20% (153)	other / not applicable: 12% (93)
main emphasis of the article on “arthroplasty”		
Yes: 33% (249)	No: 67% (517)	
main emphasis of the article on “fractures”		
Yes: 22% (171)	No: 78% (595)	

**Fig. 1:**
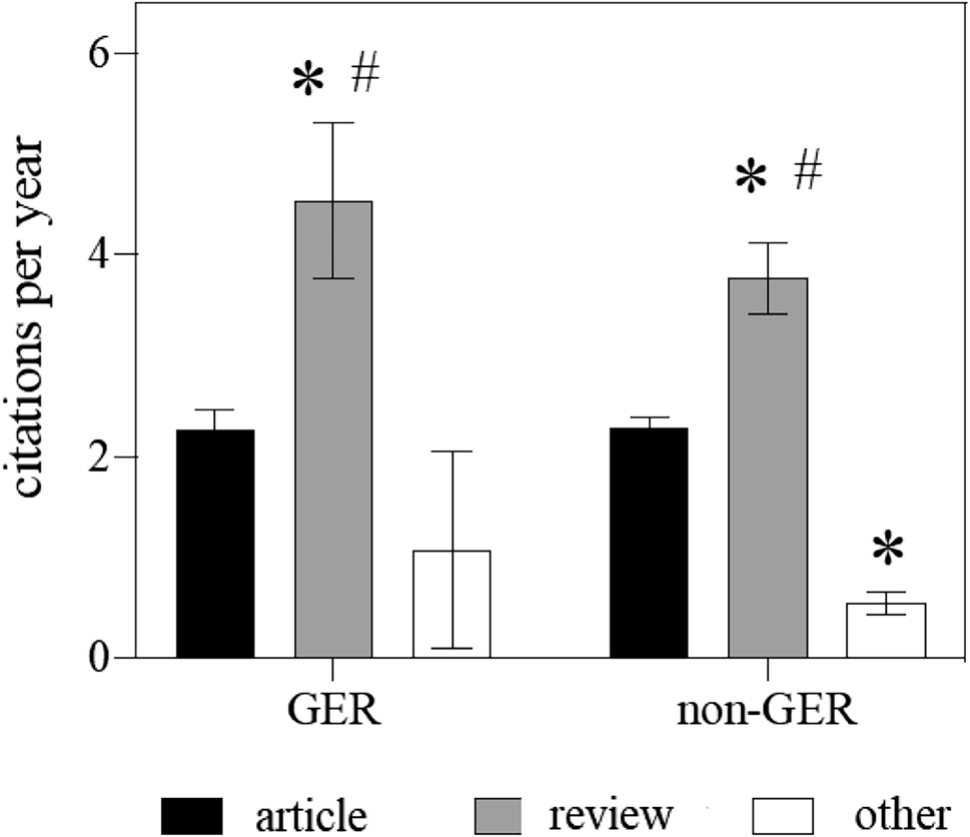
Analysis of the citations per year in the GER and non-GER group divided by the type of publication (“reviews”, “articles” or “other”). Data are given as mean ± SEM. Significant difference via 2-way ANOVA and Holm-Sidak test: *p < 0.05 vs. article; ^#^*p* < 0.05 vs. other

Data exploration for the corresponding author’s affiliation identified that 22% of all publications originate from China, 13% from France, 11% from GER, 9% from Italy, and 9% from the United States of America. We also found in 12% of all publications (94 out of 766) at least one author from GER. Only 10% of the GER publications (9 out of 94) was the corresponding author affiliated to an institute or department located outside Germany, Austria, or Switzerland. Analysis of the citations per year in the GER and the non-GER group showed that reviews were cited twice as frequently as “articles” or “other” types of publications (Fig. 1). Moreover, GER articles are equally frequently cited compared to no-GER articles (Fig. 1).

**Fig. 2:**
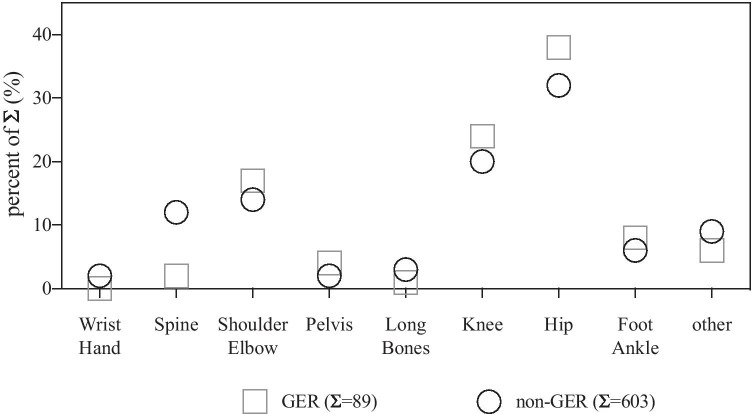
Anatomic focus of articles and reviews published for the GER and the non-GER group. Data are given in percent of total (%). Total number of each group is shown by Σ. Chi-square

Sixty-two percent of the articles and reviews of the GER publications had their main scientific focus on hip or knee, whereas 52% of the hip or knee publications accounted in total for the non-GER group. Additionally, spine-related articles and reviews were less frequently found in the GER group compared to the non-GER group. Moreover, hand and wrist publications contributed for 2% of the publications in the non-GER group, while 0 publications in this field were conducted by GER authors (Fig. 2). Similar results were observed for the corresponding author between GER and non-GER groups (Table 2).

**Table 2:** Anatomic focus of articles and reviews published for the corresponding author in the GER and the non-GER group. Data are given as percent of total (%) and as count (in parenthesis)

	corresponding author	
	non-GER	GER
Wrist & Hand	2 % (14)	0 % (0)
Spine	12 % (72)	2 % (2)
Shoulder & Elbow	14 % (83)	17 % (14)
Pelvis	3 % (16)	4 % (3)
Long Bones	3 % (19)	1 % (1)
Knee	20 % (122)	25 % (20)
Hip	32 % (195)	36 % (29)
Foot & Ankle	6 % (36)	9 % (7)
other	9 % (54)	6 % (5)

Over the last 45 years, the journal “International Orthopaedics” has published high-quality articles that significantly contributed to the advancement of orthopaedic knowledge and research [4]. The increased publication activity of Germanophone countries in the field of orthopaedic surgery has been highlighted in previous publications. A recent bibliometric analysis performed by Mavrogenis et al. confirmed that 18 of the 100 most cited articles of the past 40 years in “International Orthopaedics” originated from Germanophone countries [5]. The high scientific output of Germanophone countries can be seen as a consequence of the population size and high gross domestic product of Germany, Switzerland, and Austria and directly related to the academic performance of German-speaking universities. These parameters (population size and gross domestic product) are positively associated with orthopaedic research publication [6].

**Fig. 3:**
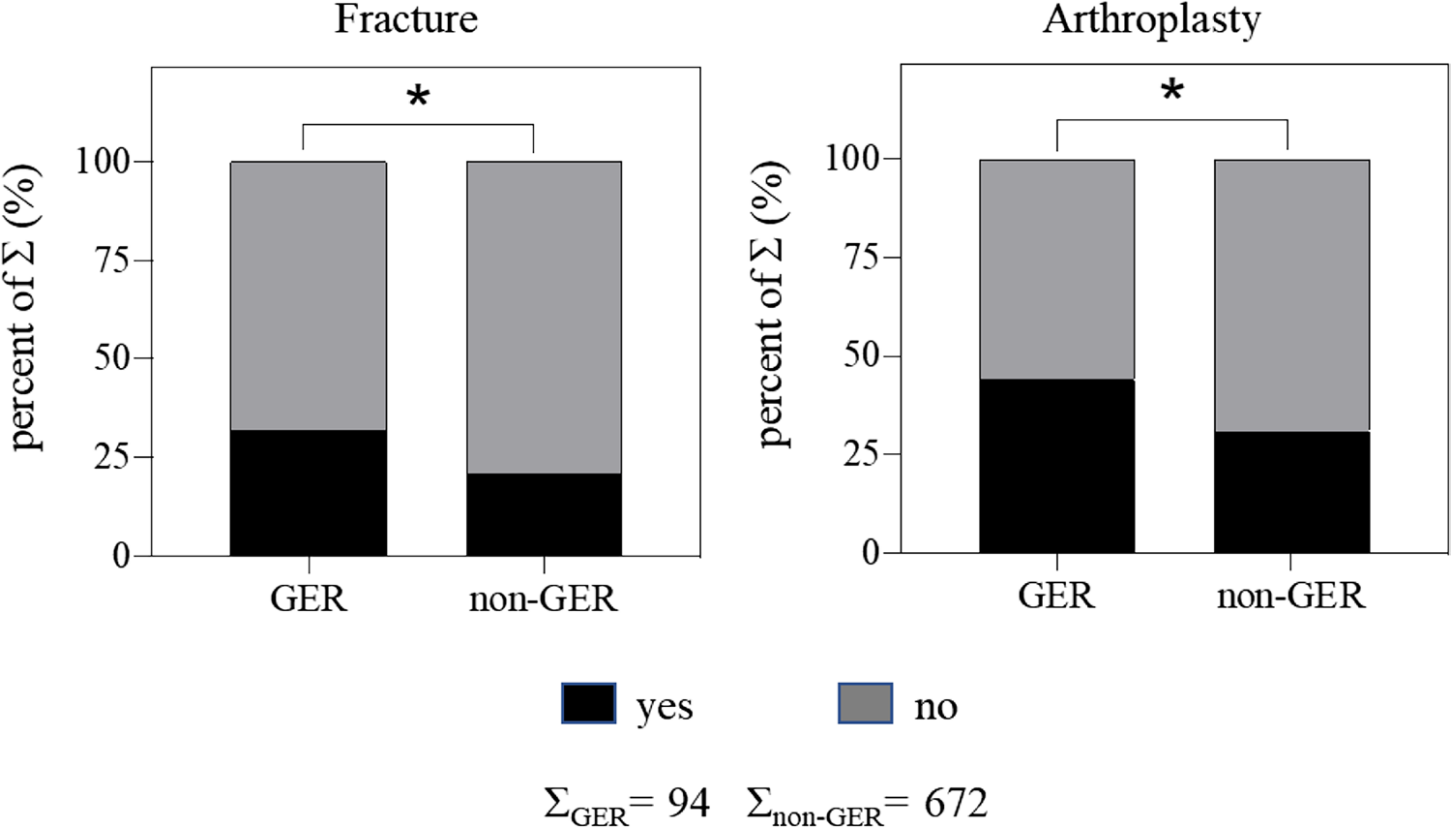
Analysis of main emphasis (fracture or arthroplasty) for the non-GER and GER group. Total number of each group is shown by Σ. Chi-square. *p < 0.05 GER vs. non-GER

The fact that reviews published in IO are cited more frequently compared to other articles indicates that the reviews are of good scientific quality. Additionally, Germanophone articles published in the IO are relevant to patient care due to the reason that the majority of these articles (62 out of 94 publications) are frequently cited clinical articles. Breakdown of the publications’ main emphasis (fracture or arthroplasty) revealed a significant difference between GER and non-GER articles. GER authors were more frequently interested in publications related to fractures or arthroplasty compared to non-GER authors (Fig. 3 and Table 3). One possible explanation for this difference between GER and non-GER groups could be based on the specialization character of Germanophone hospitals. Orthopaedic departments in Germany, Austria, and Switzerland are traditionally specialized/ divided in trauma surgery or elective orthopaedic surgery. Depending on their specialization, their research is directed more towards “fractures” or “arthroplasty”.

**Table 3:** Analysis of main emphasis (fracture or arthroplasty) for the cor-non-GER and cor-GER group. Data are given as percent of total (%) and as count (in parenthesis). Chi-square: *p < 0.05 between groups within Arthroplasty

		corresponding author	
		non-GER	GER
Fracture	no	79% (536)	69% (59)
	yes	21% (145)	31% (26)
Arthroplasty *	no	69% (469)	56% (48)
	yes	31% (212)	44% (37)

Although the quality of GER publications does not differ from publications from other countries, all GER countries have the advantage of the common German language that can be seen as a way to promote networks and drive orthopaedic research forward. One possible way that GER authors could increase their impact in IO would be to focus their research on under-represented fields like spine or hand and wrist surgery.
